# Variations in foliar monoterpenes across the range of jack pine reveal three widespread chemotypes: implications to host expansion of invasive mountain pine beetle

**DOI:** 10.3389/fpls.2015.00342

**Published:** 2015-05-19

**Authors:** Spencer Taft, Ahmed Najar, Julie Godbout, Jean Bousquet, Nadir Erbilgin

**Affiliations:** ^1^Department of Renewable Resources, University of AlbertaEdmonton, AB, Canada; ^2^Natural Resources Canada, Canadian Forest Service, Laurentian Forestry CentreQuébec, QC, Canada; ^3^Canada Research Chair in Forest and Environmental Genomics, Forest Research Centre, Université LavalQuébec, QC, Canada

**Keywords:** *Pinus banksiana*, Pinaceae, jack pine, monoterpenes, *Dendroctonus ponderosae*, mountain pine beetle

## Abstract

The secondary compounds of pines (*Pinus*) can strongly affect the physiology, ecology and behaviors of the bark beetles (Coleoptera: Curculionidae, Scolytinae) that feed on sub-cortical tissues of hosts. Jack pine (*Pinus banksiana*) has a wide natural distribution range in North America (Canada and USA) and thus variations in its secondary compounds, particularly monoterpenes, could affect the host expansion of invasive mountain pine beetle (*Dendroctonus ponderosae*), which has recently expanded its range into the novel jack pine boreal forest. We investigated monoterpene composition of 601 jack pine trees from natural and provenance forest stands representing 63 populations from Alberta to the Atlantic coast. Throughout its range, jack pine exhibited three chemotypes characterized by high proportions of α-pinene, β-pinene, or limonene. The frequency with which the α-pinene and β-pinene chemotypes occurred at individual sites was correlated to climatic variables, such as continentality and mean annual precipitation, as were the individual α-pinene and β-pinene concentrations. However, other monoterpenes were generally not correlated to climatic variables or geographic distribution. Finally, while the enantiomeric ratios of β-pinene and limonene remained constant across jack pine's distribution, (−):(+)-α-pinene exhibited two separate trends, thereby delineating two α-pinene phenotypes, both of which occurred across jack pine's range. These significant variations in jack pine monoterpene composition may have cascading effects on the continued eastward spread and success of *D. ponderosae* in the Canadian boreal forest.

## Introduction

In pine trees (genus *Pinus*), monoterpenes are a prominent class of phytochemicals that play a significant role in tree-insect interactions (Phillips and Croteau, 1999; Franceschi et al., 2005; Raffa et al., 2005, 2013; Moore et al., 2014). Generally, monoterpenes are a central aspect of pines' constitutive and inducible defenses and are an essential component of pine defensive resins that are toxic to many herbivorous insects, including subcortically-feeding bark beetles (Coleoptera: Curculionidae, Scolytinae) (Raffa et al., 1985, 2005; Cates et al., 1987; Phillips and Croteau, 1999; Keeling and Bohlmann, 2006). The influence of pine monoterpenes on bark beetles is critical as about 500 species of these sub-cortical herbivorous insects feed on pine trees, including many tree species of significant ecological and economic importance (Wood, 1982; Bentz et al., 2010; Safranyik et al., 2010; Raffa et al., 2013). Moreover, various aspects of beetle biology, such as dispersal, host selection and colonization, physiology and behavior are strongly influenced by host monoterpenes (e.g., Raffa et al., 2005, 2013; Seybold et al., 2006).

There are numerous means by which monoterpene composition of host trees mediates and influences bark beetle-host interactions. However, the relationship between monoterpenes and bark beetles is not simple or one sided, as, despite their toxic properties, many monoterpenes can attract or otherwise benefit bark beetles (Chénier and Philogène, 1989; Erbilgin and Raffa, 2000a,b; Seybold et al., 2006; Blomquist et al., 2010). For example, the most abundant monoterpenes of pines, such as α-pinene, β-pinene, myrcene, limonene, camphene, and 3-carene can all act as attractants for pine engraver beetles (*Ips grandicollis*) (Werner, 1972; Chénier and Philogène, 1989; Erbilgin and Raffa, 2000a,b). Furthermore, host plant volatiles can attract bark beetle predators or mediate predator attraction to bark beetle pheromones (e.g., Erbilgin and Raffa, 2001).

In addition to directly attracting beetles, certain host monoterpenes can act as precursors to pheromone components of some bark beetle species, best exemplified by biosynthesis of the pheromone constituents verbenol, verbenone, and verbenene from host derived α-pinene (reviewed by Blomquist et al., 2010). This specific metabolic process is of particular importance to the mountain pine beetle (*Dendroctonus ponderosae*) as this pathway is the production means for the primary aggregation pheromone of the female beetles (Blomquist et al., 2010). Upon initial infestation, female *D. ponderosae* hydroxylate the host monoterpene α-pinene into their primary aggregation pheromone, *trans*-verbenol. This compound is essential for attraction of male and female beetles and successful aggregation and thus successful reproduction (Safranyik et al., 2010). Following their arrival, male *D. ponderosae* produce another aggregation pheromone, *exo*-brevicomin, which is synthesized *de novo* by epoxidation and cyclization of its precursor long-chain fatty acids and acts synergistically with *trans*-verbenol to attract enough conspecifics to overwhelm tree defenses (Pureswaran et al., 2000). When the beetles approach their optimum colonization density on host trees, males produce the anti-aggregation pheromone frontalin from monoterpenoid or long-chain fatty acid precursors to maintain the most favorable beetle density. Finally, the anti-aggregation pheromone verbenone is thought to be produced through an auto-oxidation of the host monoterpene α-pinene by both sexes. This close relationship between host secondary chemistry and *D. ponderosae* is of particular interest because host secondary chemistry can affect herbivorous insects' host shifts (Jermy, 1984), which *D. ponderosae* has recently experienced (Erbilgin et al., 2014).

First confirmed in 2011, *D. ponderosae* has expanded its range from lodgepole pine (*Pinus contorta*)-dominated forests into jack pine (*Pinus banksiana*)-dominated stands in the boreal forest in western Canada (Alberta), indicating a range and host shift (Cullingham et al., 2011). This is an ecological and economic concern, as jack pine is distributed through the boreal forest from Alberta to the Atlantic coast, representing a 4000 km corridor between western and eastern pine species. There are numerous hypotheses addressing how various factors can affect an herbivorous insect's host shift, but the importance of the host's secondary chemistry to the insect is significant (Jermy, 1984; Feeny, 1991; Becerra, 1997; Murphy and Feeny, 2006; Erbilgin et al., 2014).

The complex relationship between host chemistry and *D. ponderosae* affects beetle physiology and ecology and illustrates the importance of understanding the phytochemical landscape of the beetles' novel host, jack pine. Considerable variation in other conifer monoterpene profiles has resulted in the classification of multiple intraspecific chemical phenotypes, or chemotypes. For example, lodgepole pine monoterpenes were shown to persist in three common chemotypes defined by different proportions of β-phellandrene and β-pinene as well as five rare chemotypes (Forrest, 1981). Furthermore, induced monoterpenes in lodgepole pine varied between trees sampled in northern and southern British Columbia, indicating chemically disparate populations (Clark et al., 2014). Similarly, monoterpene profiles of ponderosa pine (*P. ponderosae*), another host of *D. ponderosae*, can be classified into three discrete chemotypes based on 3-carene, α-pinene and β-pinene proportions (Latta et al., 2003; Davis and Hofstetter, 2012). Additionally, Scots pine (*P. sylvestris*) monoterpene profiles are categorized into chemotypes based on the presence of 3-carene, with subsequent variation in concentrations of α-pinene, β-pinene, and camphene (Thoss et al., 2007). Such variations in pine monoterpene chemistry can influence bark beetle activities, including maternal gallery excavation, fecundity, survivorship, fitness, and pheromone production (Boone et al., 2011; Lusebrink et al., 2011; Reid and Purcell, 2011; Davis and Hofstetter, 2012; Erbilgin et al., 2014). Additionally, pine chemotypes affect growth performance of obligate fungal symbionts of bark beetles (Davis and Hofstetter, 2012).

However, causes underlying variation of pine monoterpene chemistry are not entirely understood and effects of genetic and environmental factors on monoterpene composition can be variable. For example, in Scots pine, individual monoterpenes appear to have different levels of broad sense heritability, as 3 carene, myrcene, limonene and terpinolene tend to be primarily genetically controlled, whereas α-pinene and β-pinene depend more on environmental factors (Baradat and Yazdani, 1988). Similarly, in lodgepole pine inoculated with fungus *Grosmannia clavigera* associated with *D. ponderosae*, environment affects the induction of certain monoterpenes while showing no effect on others, thereby suggesting genetic control of certain, but not all, monoterpenes (Ott et al., 2011). The varied influences that genetic and environmental factors have on monoterpene composition and the diverse and extensive means by which host chemistry affects bark beetles illustrate the importance of exploring the phytochemical landscape of jack pine. Furthermore, defining chemotypes which may variably affect the ecology and survival of *D. ponderosae* in jack pine forests could provide foresight into the beetle's continued eastward spread.

Due to importance of monoterpenes in bark beetle biology and ecology (reviewed by Raffa et al., 2005), in our investigations we focused on monoterpene composition of jack pine in both natural and provenance stands. Our study aims to first determine whether jack pine exhibits different chemotypes based on overall monoterpene proportions throughout the boreal forest. Second, we determine how jack pine's monoterpene composition varies with climatic factors and how such variation relates to chemotype frequency. Finally, we investigate whether enantiomeric ratios of major chiral monoterpenes vary among different populations of jack pine throughout the boreal forest and establish whether such variation can be classified into different phenotypes based on enantiomeric composition.

## Experimental

### Sampling

All needle samples had been previously used for genetic diversity analysis at the DNA level in Godbout et al. (2005, 2010). Needles were collected during the active growth period from a total of 601 jack pine trees across the north eastern range of jack pine in Canada and the United States. Of these, 231 trees were from natural stands, representing 25 locations, with 6–10 trees per location. The remaining 369 trees were collected from four provenance trials in Petawawa (Ontario), Ste-Christine-d'Auvergne and Fontbrune (Quebec), and Dubee Settlement (New Brunswick), representing 38 provenance locations with 6–10 trees sampled per provenance. Trees were 28-37 years old. After collection, samples were stored at −25°C until they were packed in dry ice and shipped from Laval University to the University of Alberta, at which time they were stored at −40°C.

### Tissue extracts

Needle tissue was ground in liquid nitrogen and 100 mg of the tissue were transferred to a 1.5 ml microcentrifuge tube where samples were extracted twice with 0.5 ml methyl tert-butyl ether solvent with 0.002% tridecane as an internal standard. After adding solvent, samples were vortexed at 3000 rpm for 30 s, sonicated for 10 min and centrifuged at 13,000 rpm and 0°C for 15 min. Extracts were transferred into amber GC vials and stored at −40°C until analysis.

### GC-MS analysis

Monoterpenes extracted were analyzed using similar methods reported in Erbilgin et al. (2014). Briefly, extracts (1 μl) were analyzed using a GC-MS (7890A/5062C, Agilent Tech, Santa Clara, CA, USA) equipped with a chiral column (HP Innowax-20B column (ID 0.25 mm, length 30 m); Agilent Tech) with helium as the carrier gas flow set to 1.1 ml min^−1^. Each analysis began at an initial temperature of 75°C for 15 min, followed by an increase in 5°C per min until 230°C was reached. Peaks were identified using the following standards: Borneol, pulegone, α-terpinene, γ-terpinene, α-terpineol, 3-carene, terpinolene, α- and β-thujone, (−)-α-pinene, (+)-α-pinene, (−)-β-pinene, (+)-β-pinene, (−)-limonene, (+)-limonene, (−)-camphene, (+)-camphene, sabinene hydrate, myrcene, p-cymene, *cis*-ocimene (SAFC Supply Solutions, St. Louis, MO, USA), and β-phellandrene (Glidco Inc., Jacksonville, FL, USA). Chemical purity of all these compounds was higher than 99%. Compounds were identified by comparing retention times and mass spectra to those of the standard chemicals. Quantity of chemicals was calculated using response curves generated from analyses of a dilution sequence of known quantities of standards. Calibration with these standards allowed for analysis of quantitative differences on monoterpene concentrations among samples. The amount of monoterpenes extracted per wet weight of needle (μg mg^−1^) was reported.

### Statistical analyses

Given initial analyses that suggested monoterpene composition is at least partially influenced by environmental conditions, all statistical analyses treated samples from natural and provenance stands separately. Direct comparisons using both stand types were not done unless stand type did not affect the variable in question. All statistical analyses on monoterpene concentration and proportion data was performed in R statistical program version 2.15.0 (R Development Core Team, 2013) using the ecodist (Goslee and Urban, 2007), mvpart (Therneau et al., 2013), vegan (Oksanen et al., 2013), and pvclust (Suzuki and Shimodaira, 2006) packages. Proportions were determined by dividing the concentration of an individual monoterpene (ug mg^−1^) by the sum of all monoterpene concentrations.

Hierarchical cluster analyses were performed separately on samples from natural and provenance stands to establish chemotypes based on overall similarities in monoterpene proportions of individual trees. The hierarchical cluster analysis uses a distance matrix to cluster samples in a hierarchical structure based on overall similarity, moving from broad to specific similarities. The Bray-Curtis distance measure was used to generate a distance matrix. Cluster analyses were each subjected to a bootstrap re-sampling analysis to generate approximately unbiased *p*-values with each cluster of interest. The cluster analysis was trimmed to the three broadest groups to be used as chemotypes, as these represented broad similarities between trees. In turn, these chemotypes were used as discrete, manipulated variables to determine variance explained by each and bar plots of the standardized data were generated to visualize monoterpenes driving these divisions. Additionally, a similar cluster analysis was trimmed to two groups and used to classify trees into different trends observed in α-pinene enantiomer composition.

Non-metric multidimensional scaling (NMDS) was used to visualize the relationship between chemotypes and monoterpene proportions as well as climatic variables and monoterpene concentrations. Climatic variables considered were mean annual precipitation, degree-days of 0°C and continentality (temperature difference between warmest and coldest months). Values were attained through the software package ClimateNA (v4.85) and represent monthly data over a 30 year period (1961–1990) generated by the Parameter Regression of Independent Slopes Model (PRISM) (Hamann et al., 2013). The Bray-Curtis distance measure was used to create a dissimilarity matrix from which an NMDS ranks the distances between samples. These distances were then represented in a two-dimensional configuration minimizing stress, which is a metric of how well the configuration plots similar points close together. Two vectors with angles that are less than 90° represent positively correlated variables while angles greater than 90° represent negative correlations and vector length corresponds to the strength of the variable.

Linear regression analyses were performed to determine Pearson's correlation coefficient (r) values between climatic variables and monoterpene concentrations. Correlations were considered strong if *r* > 0.7, moderate if 0.7 > *r* > 0.5, and weak if *r* < 0.5. Additionally, for trees sampled from natural stands, correlations between climatic variables and the proportion of chemotypes occurring at individual sites were determined.

In order to test how the two enantiomers of α-pinene were distributed among trees, a permutational multivariate analysis of variance (per MANOVA) test was used to determine differences between observed α-pinene phenotype groups. Groups were defined by a hierarchical cluster analysis. A per MANOVA test assesses the ratio of distances between points within groups and across groups. For this, the Bray-Curtis distance measure was used. The class variable (phenotype group) is permutated multiple times to establish a distribution of the test statistic, thereby eliminating any assumptions about normality. Finally, the test statistic is compared to the newly generated probability distribution to determine a *p*-value.

## Results

### Jack pine chemotypes

To define overall monoterpene chemotypes, we had an *a priori* focus on the monoterpenes (−) and (+) α-pinene, (−) and (+) β-pinene, (−) and (+) limonene, 3-carene, myrcene, and terpinolene because of their biological and ecological relevance to bark beetles as well as their prominence in jack pine (>95% of total monoterpenes) (Raffa et al., 2005; Colgan and Erbilgin, 2011; Lusebrink et al., 2011; Erbilgin and Colgan, 2012; Clark et al., 2014; Erbilgin et al., 2014). Additionally, we found these compounds defined overall monoterpene composition trends in our data well. The two enantiomers of each β-pinene and limonene were grouped together because they were very closely correlated (*r* > 0.99) and maintained at a constant ratio. However, the (−):(+) α-pinene ratio varied and exhibited different trends throughout jack pine's range so the α-pinene enantiomers were examined as individual compounds. Because our data suggests that monoterpene concentrations are correlated with climatic variables, evaluations of chemotypes were done separately in natural and provenance stands. Additionally, minor variations between stand types were observed and analyzing data by stand types allowed us to discern genetic vs. environmental effects on monoterpene composition. The complete monoterpene profiles of trees were reported in Supplementary Table 1.

**Figure 1:**
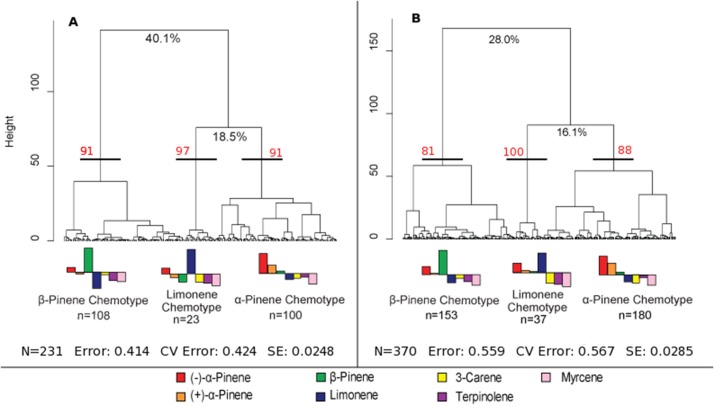
**Hierarchical cluster analyses of jack pine (*Pinus banksiana*) monoterpene proportions in (A) natural stands across eastern Canada and northeastern U.S. and (B) provenance stands representing jack pine populations from Alberta to the Atlantic coast**. The three broadest divisions of each cluster analysis were used to define chemotypes. The percent values at the first two divisions of each cluster analysis represent the variance explained at those divisions. Approximately unbiased bootstrap values are indicated in red for each chemotype. The error is the remaining variance that is not explained by the first two divisions, the CV error is the cross validated error and SE is the standard error. The bar plot represents normalized monoterpene proportion data and the bars above the mid-line represent monoterpene proportions that are the bases for group divisions.

**Figure 2:**
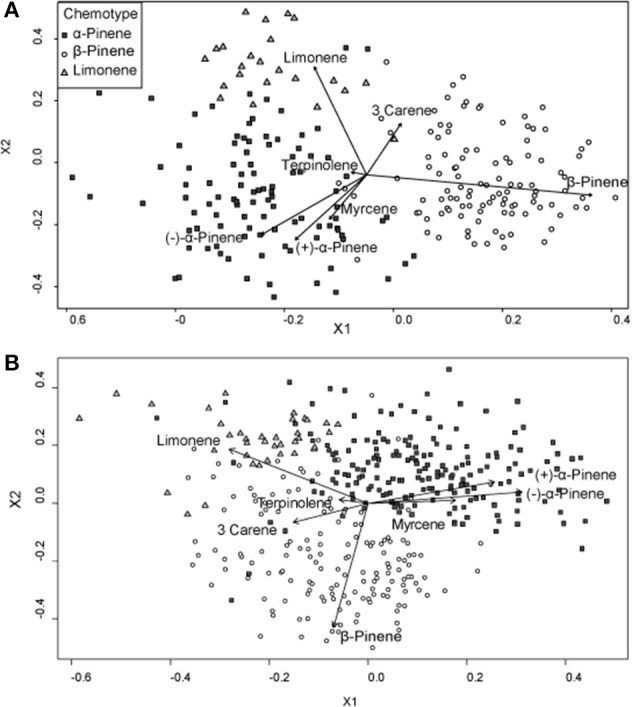
**Non-metric multidimensional scaling (NMDS) plots of monoterpene proportions of jack pine (*Pinus banksiana*) in (A) natural stands and (B) provenance stands**. Points are divided by chemotypes defined by a hierarchical cluster analyses performed on samples from each respective stand type. Vectors represent individual monoterpene proportions. Each point represents an individual tree.

A hierarchical cluster analysis of monoterpene proportions in jack pines from natural stands established three broad groups of trees based on overall similarities of monoterpene proportions (Figure 1A). These three groups were best defined by relatively high proportions of β-pinene (108 trees), α-pinene (100 trees), and limonene (23 trees) and indicate three distinct chemotypes (Table 1). Despite variations in the (−):(+) α-pinene ratio, high proportions of both enantiomers were closely associated with the same chemotype, leading it to be classified as simply α-pinene chemotype. These chemotypes explained a total of 58.6% of the observed variance in monoterpene proportions. The division between the β-pinene chemotype and the other two chemotypes was the most distinct and explained 40.1% of monoterpene proportion variance, while the remaining division between the α-pinene and limonene chemotypes explained 18.5% of variance. An NMDS of monoterpene proportions from natural stands showed that individual trees grouped by the three chemotypes were closely associated with their respective defining monoterpene vectors (Figure 2A).

**Table 1:** **Approximate defining properties of the three chemotypes derived from a hierarchical cluster analysis of jack pine (*Pinus banksiana*) trees in (a) natural stands in populations of eastern Canada and northeastern U.S.A., and (b) provenance stands representing jack pine populations from Alberta to the Atlantic coast**.

**Chemotypes**	**Defining monoterpene trends (% in the total monoterpenes)**
**NATURAL STANDS**	
α-Pinene	(−) and (+) α-Pinene > 20%, β-Pinene < 20%, Limonene < 20%
β-Pinene	β-Pinene > 24%, (−) and (+) α-Pinene < 18%, Limonene < 20%
Limonene	Limonene > 20%
**PROVENANCE STANDS**	
α-Pinene	(−) and (+) α-Pinene > 15%, β-Pinene < 15%, Limonene < 10%
β-Pinene	β-Pinene > 15%, (−) and (+) α-Pinene < 15%, Limonene < 20%
Limonene	Limonene > 15%, α-Pinene < 15%, β-Pinene < 15%

Furthermore, a hierarchical cluster analysis of trees sampled from provenance stands maintained these chemotypes (Figure 1B) and an NMDS showed similar chemotype divisions to that of trees sampled from natural stands (Figure 2B). This cluster analysis was also compared to genetic information known about these specific samples from Godbout et al. (2005, 2010). However, current monoterpenes showed no correlations to phylogeographic history, genetic lineages, provenance location, provenance climate, phenology or ontogeny. Rather, the broadest divisions of monoterpene proportions from provenance stands aligned with the monoterpene proportions of the chemotypes observed in natural stands. Once again, the β-pinene chemotype was widely separated from the other chemotypes while the α-pinene and limonene chemotypes were distributed more closely to each other, which is in accordance with what was observed in the hierarchical cluster analysis. However, in provenance stands, the relative numbers of trees in each group changed, as the α-pinene chemotype consisted of the most trees, with 180, while the β-pinene chemotype had 153 trees and limonene chemotype included 37 trees.

**Figure 3:**
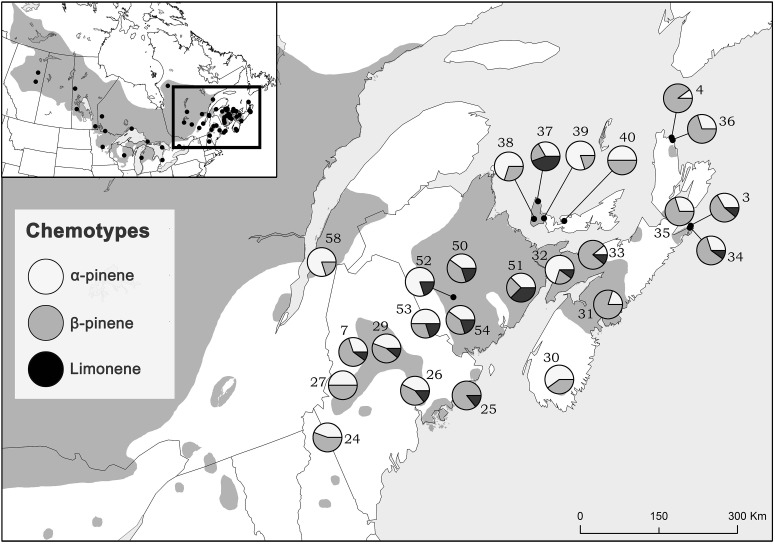
**Jack pine (*Pinus banksiana)* natural stand locations in eastern Canada and northeastern US colored proportionately by chemotype frequency**. Numbers represent site designations and shaded area of map represents jack pine's range.

Considering trees from natural stands, all three chemotypes occurred across the sampled natural range, frequently all persisting within the same site while no site was exclusively any single chemotype (Figure 3). Interestingly, the β-pinene and the α-pinene chemotypes were present in 96 % and the limonene chemotype was present in 56% of sites. Additionally, climatic variables were correlated to chemotype proportion at individual sites (Table 2) and therefore chemotypes of trees from provenance stands could not be plotted on a map, as all were grown in the same environment which would have a homogenizing effect on chemotype. For example, the proportion of the α-pinene chemotype was negatively correlated with mean annual precipitation, but was positively correlated with degree-days above 0°C (Table 2). The proportion of the β-pinene chemotype was negatively correlated with continentality and degree-days above 0°C, but was positively correlated with mean annual precipitation (Table 2).

**Table 2:** **Pearson correlation coefficient values (*r*) between climatic variables and chemotype frequencies from natural stands of jack pine (*Pinus banksiana*) populations in Canada and northeastern U.S.A**.

**Chemotypes**	***r*-values for chemotype frequencies as a function of climate**			
	**Longitude**	**TD**	**MAP**	**DD0**
α-Pinene	−0.28	0.36	−0.41^*^	0.42^*^
β-Pinene	0.31	−0.46^*^	0.51^*^	−0.44^*^
Limonene	−0.098	0.24	0.23	0.10

*TD, Continentality (Temperature Differance between warmest and coldest month); MAP, Mean Annual Precipitation; DD0, Degree-Days above 0°C*.

* *indicates P < 0.05. Significant r-values are in bold*.

### Individual monoterpene variation

Climate variables affected concentrations of individual monoterpenes differently. In natural stands, both enantiomers of β-pinene and α-pinene showed significant correlations with all climate variables (Table 3). Myrcene, 3-carene, terpinolene and both enantiomers of limonene were not correlated to any climate variables, with the exception of a weak correlation between 3-carene and continentality (Table 3). Of any correlation between climate factors and monoterpene concentrations, mean annual precipitation had the strongest positive correlation with β-pinene and α-pinene, whereas continentality was most strongly negatively correlated with the same two monoterpenes (Table 3). In provenance stands, there were no significant correlations between monoterpene concentrations and climate variables from provenance origin with the exception of a weak correlation between mean annual precipitation and terpinolene and myrcene (Supplementary Table 2).

**Table 3:** **Pearson correlation coefficient values (*r*) between climatic variables and individual monoterpene concentrations in natural jack pine (*Pinus banksiana*) stands in eastern Canada and northeastern US**.

**Monoterpenes**	**Longitude**	**TD**	**MAP**	**DD0**
(−)-α-Pinene	0.4422^*^	−0.54901^*^	0.741^*^	−0.41917^*^
(+)-α-Pinene	0.51^*^	−0.56293^*^	0.7^*^	−0.45916^*^
(−)-β-Pinene	0.6952^*^	−0.74141^*^	0.8197^*^	−0.6544^*^
(+)-β-Pinene	0.6785^*^	−0.74031^*^	0.8177^*^	−0.65321^*^
(−)-Limonene	0.2411	−0.05026	0.0801	−0.07606
(+)-Limonene	0.2244	−0.04071	0.0669	−0.06793
	0.4573^*^	−0.37397^*^	0.2556	−0.27614
Myrcene	0.247	−0.21444	0.3195	−0.1415
Terpinolene	0.1562	−0.07907	0.0767	−0.0766

*TD, Continentality (Temperature Differance between warmest and coldest month), MAP, Mean Annual Precipitation, DD0, Degree-Days above 0°C*.

* *indicates *P* < 0.05. Significant r-values are in bolded*.

Because environmental factors affected individual monoterpene concentrations differently, monoterpene proportions of the total profile were not constant and thus it was difficult to elucidate overall trends between monoterpene proportions and climate. For example, β-pinene and α-pinene concentrations increased with increasing mean annual precipitation, while limonene concentrations remained unaffected, meaning that limonene's proportion of the total monoterpene profile decreased. Generally, though, climatic variables were less strongly correlated to monoterpene proportions than to concentrations. Finally, in both natural and provenance stands, (−) and (+)-α-pinene and myrcene proportions were positively correlated to each other, but were negatively correlated to β-pinene, limonene and 3-carene (Figure 2).

### Enantiomeric composition

**Figure 4:**
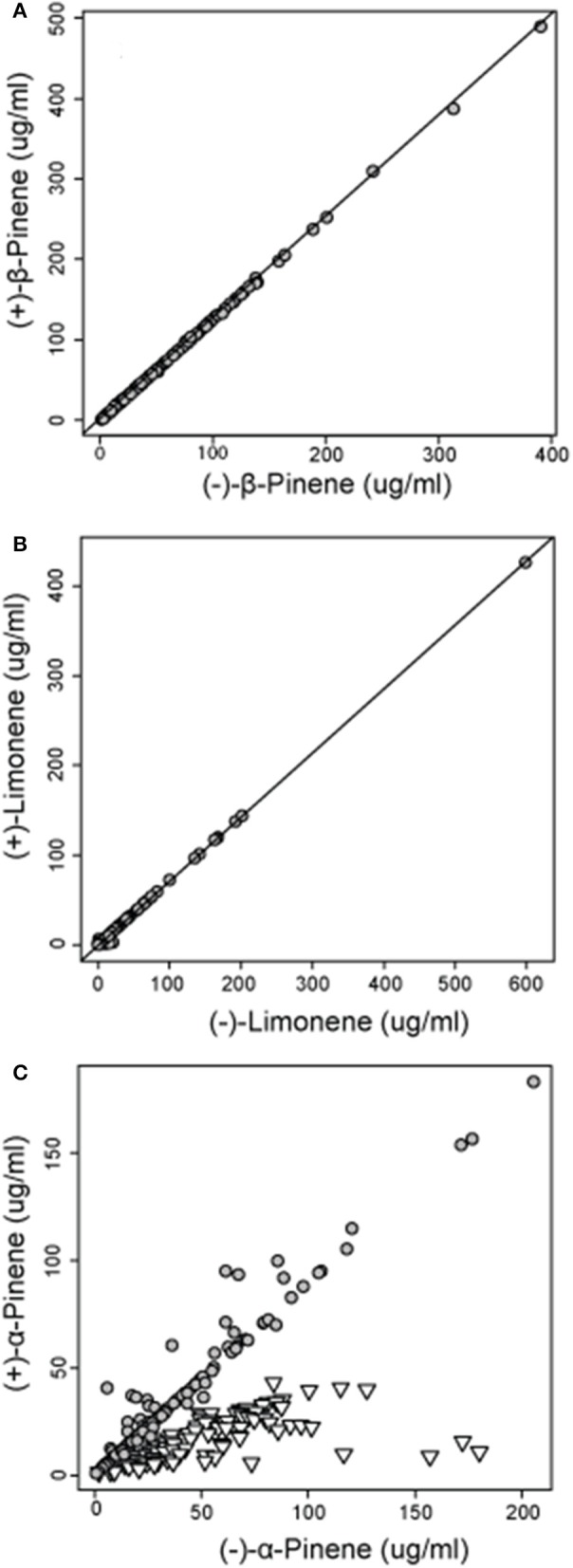
**Concentrations of (A) (−) and (+) β-pinene (*r* = 0.999) and (B) (−) and (+) limonene (*r* = 0.999) in natural jack pine (*Pinus banksiana*) stands plotted against each other to illustrate the relative amount of each enantiomer**. For concentrations of **(C)** (−) and (+) α-pinene, gray circles represent group 1 and inverted triangles represent group 2, both as defined by a hierarchical cluster analysis.

Analyses of the chiral monoterpenes α-pinene, β-pinene and limonene showed that enantiomeric composition trends varied between individual compounds. While (−):(+)-β-pinene and (−):(+)-limonene remained at constant ratios in all sampled trees across jack pine's range, (−):(+) α-pinene exhibited two separate trends, thereby delineating two distinct phenotypes (Figure 4). The separation of these two distinctive α-pinene phenotypes was supported by a hierarchical cluster analysis which divided trees into two broad groups (group 1 and group 2) depending on (−):(+) α-pinene ratios (Supplementary Image 1). These groups corresponded to the two distinct trends observed in (−):(+)-α-pinene ratio (Figure 4). The enantiomeric composition patterns observed for α-pinene, β-pinene and limonene were maintained in natural and provenance stands, though stand types were analyzed separately.

**Figure 5:**
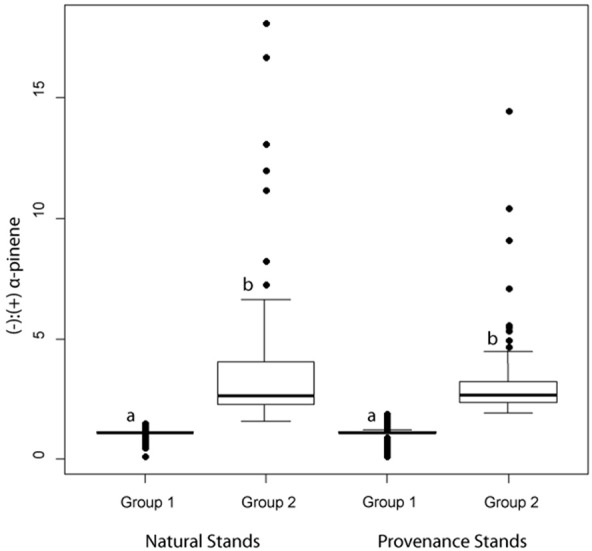
**Box and whisker plots of jack pine (*Pinus banksiana*) α-pinene groups as defined by a hierarchical cluster analysis**. Boxes represent data between the first and third quartiles while the whiskers represent the range of data excluding outliers, represented by dots. Letters represent statistically significant differences at α = 0.05.

In natural stands, α-pinene phenotype group 1 consisted of 128 trees and had a mean (−):(+) ratio of 1.11 (± 0.22) (Figure 5). Group 2 consisted of 103 trees and had a mean (−):(+)-α-pinene ratio of 3.48 (± 2.77) (Figure 5). In provenance stands, group 1 consisted of 249 trees and had a mean (−):(+) α-pinene ratio of 1.09 (± 0.29) and group 2 included 120 trees with a mean (−):(+) α-pinene ratio of 3.11 (± 1.61) (Figure 5). A perMANOVA test showed that groups 1 and 2 vary significantly [*F*_(*1, 25.5*)_ = 983, *P* = < 0.001] while there wasn't any difference between natural and provenance stands [*F*_(*1, 0.04*)_ = 1.36, *P* = 0.24].

**Figure 6:**
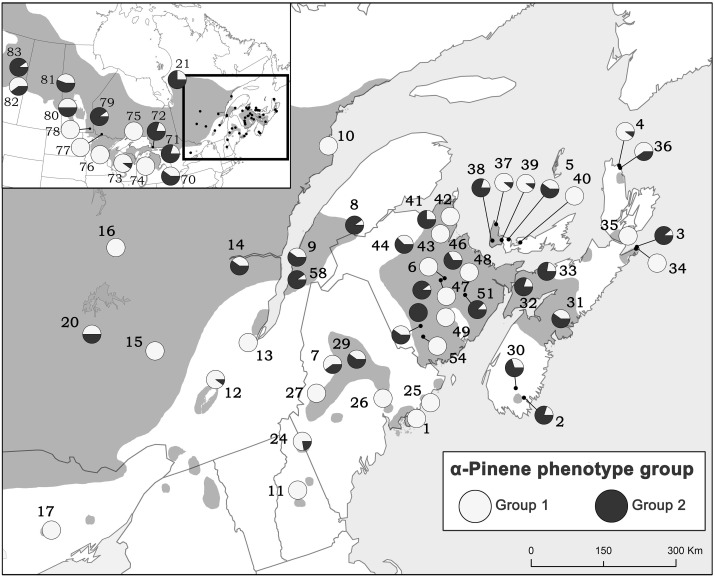
**The proportion of trees exhibiting each of the α-pinene phenotypes from all natural stands and provenance origins**. Group 1 has a mean (−):(+) α-pinene ratio of 1.10 (±0.25) and group 2 has a mean (−):(+) α-pinene ratio of 3.37 (±2.24). Numbers represent site designations and shaded area of map represents jack pine's range.

Due to the importance of α-pinene to pheromone production by *D. ponderosae*, we also evaluated the proportion of trees exhibiting each of the α-pinene phenotypes from all natural stands and provenance origins (Figure 6). Although both groups occur throughout jack pine's range, it appears that jack pine is dominated by lower (−):(+)-α-pinene ratios (group 1).

## Discussion

### Jack pine chemotypes

We defined three distinct chemotypes in jack pine most notably characterized by relatively high proportions of three monoterpenoid compounds, α-pinene, β-pinene, and limonene. These three chemotypes were maintained in both natural and provenance stands and can co-occur in the same locality. This indicates a strong genetic influence on chemotype formation in jack pine, as monoterpene proportions did not simply conform to their environment, but rather persisted as a chemically heterogeneous forest, supporting earlier conclusions that pine monoterpenes are in part genetically controlled (Hanover, 1966, 1971; Forrest, 1981; Davis and Hofstetter, 2012; Clark et al., 2014).

However, despite an apparent genetic influence on chemotype formation, chemotypes were not correlated to jack pine's phylogeographic history or historical genetic lineages as defined in Godbout et al. (2005, 2010). Through evaluation of maternally inherited (i.e., seed-dispersal) mitochondrial DNA minisatellite markers, Godbout et al. (2010) defined five jack pine genetic lineages and a genetically distinct population isolated on Canada's east coast, likely arising after the last glacial maximum about 21,000 cal BP. Nonetheless, these genetic groups apparently have no or minimal effect on monoterpene expression, as we consistently observed no monoterpene composition patterns corresponding to these genetic lineages. A possible explanation for the lack of correlations between genetic lineages and monoterpene composition is that paternally inherited (i.e., pollen-dispersal) chloroplast DNA has a relatively uniform distribution across jack pine's range (Godbout et al., 2010). Therefore, jack pine's widely dispersed pollen may be genetically homogenizing, thus potentially eliminating phenotypic differences in monoterpene expression correlated to historic genetic lineages. Conversely, genetic influences on chemotypes and monoterpene composition may be due to adaptations or factors unrelated to the phylogeographic and genetic factors considered in this study.

Moreover, in addition to evidence suggesting a genetic influence on jack pine chemotypes, we detected weak to moderate correlations between chemotype distribution and some climate variables, thereby demonstrating that chemotype formation is influenced by both genetic and environmental factors. This may explain why chemotype divisions were less pronounced in provenance stands, as these trees would be subjected to the same environment which would have somewhat of a homogenizing effect on monoterpene proportions. Additionally, it should be noted that limitations to our study, including tree ages, may obfuscate correlations between monoterpenes and other factors, such as genetic lineages, though neither ontogeny nor phenology explained monoterpene proportions in the current study.

The observed heterogeneous monoterpene chemotype distribution also demonstrates that there is no single homogenizing selective pressure favoring one chemotype over the others as chemical polymorphism in plants is critical for reciprocal natural selection between plants and herbivorous insects (Raffa and Berryman, 1987; Becerra et al., 2009; Iason et al., 2011; Davis and Hofstetter, 2012; Moore et al., 2014). Because monoterpenes are primarily involved in conifer defenses against biotic agents (Phillips and Croteau, 1999; Wallin and Raffa, 1999, 2001; Raffa et al., 2005, 2013; Erbilgin et al., 2006; Colgan and Erbilgin, 2011), there would be strong selection for one chemotype if it was superior or favorable in defending against attacking agents under all circumstances. Alternatively, each chemotype may provide better defenses against specific attackers, as monoterpene effects can vary between attacking guilds (Wallin and Raffa, 2001; Raffa et al., 2005).

### Climate, monoterpenes, and chemotypes

In the current study, jack pine oleoresin shows substantial variation in its monoterpene concentrations across its range. Although the exact mechanism of such variation is not clear, we demonstrated that some climatic factors, such as continentality and mean annual precipitation, are correlated with certain monoterpene concentrations in natural stands and that individual monoterpenes responded differently to different climatic variables.

While all climatic variables were significantly correlated with concentrations of both enantiomers of α-pinene and β-pinene, concentrations of (−) and (+)-limonene, terpinolene, and myrcene were not at all related to climate. The monoterpene concentrations not correlated to climate were also not correlated to jack pine's distribution, indicating environment has no effect on them. Finally, 3-carene concentration varied somewhat with environment, as it was significantly correlated to continentality and longitude, signifying that it changes across jack pine's distribution in part due to environmental variables that are not measured in the current study. In general, these findings demonstrate that individual monoterpenes in jack pine are controlled by different mechanisms and are variably influenced by genetic and/or environmental factors. Some monoterpenes, specifically α- and β-pinene, display phenotypic plasticity and change with climate, while others, such as limonene, terpinolene, and myrcene do not change with environment and are therefore likely under genetic control. Overall, these results are in agreement with previous studies which have reported that limonene, myrcene and 3-carene are strongly heritable while α-pinene and β-pinene are more dependent on environmental factors (Baradat and Yazdani, 1988).

Interestingly, mean annual precipitation, associated with favorable growing conditions, was positively correlated with monoterpene concentrations. Conversely, continentality, associated with more harsh abiotic conditions, was negatively correlated with monoterpene concentrations. This is similar to previous findings that have indicated overall pine secondary metabolite levels were higher in environments with reduced climate related abiotic stress, such as drought (Wallis et al., 2011). Not only would such climates decrease abiotic limitations to a tree's physiology, but they would also increase pest development, thereby increasing selective pressure on trees to produce greater defense compounds, such as monoterpenes (Wallis et al., 2010, 2011).

The observed environmental influence on only some monoterpene concentrations and lack of influence on others show that both environment and genetics affect jack pine chemotypes. This was supported by the positive correlations between the proportion of β-pinene chemotype trees at individual sites and climate variables that were also positively correlated to β-pinene concentration, such as mean annual precipitation. Because β-pinene concentration was affected by climate more strongly than any other monoterpene, the proportion of trees of its chemotype is also most closely associated with the same climate variables in the same directions. In contrast, the proportion of α-pinene chemotype trees at individual sites exhibited the opposite directional relationship to climate variables than α-pinene concentrations. This demonstrates that, because β-pinene concentrations were more strongly correlated to climate variables than were α-pinene concentrations, changes in the proportion of trees of the β-pinene chemotype came at the expense of trees of the α-pinene chemotype. Finally, despite no correlation with climate, the limonene chemotype persisted in the same sites with the other chemotypes, showing that climate did not equally influence the variation of all chemotypes, thereby indicating a genetic effect on chemotype.

### Enantiomeric composition

In our jack pine trees, enantiomeric ratios of β-pinene and limonene were canalized and did not vary across jack pine's range or between natural and provenance stands. However, (−):(+) α-pinene ratios showed two distinct trends qualifying two phenotypes (group 1 and group 2). Both groups had (−) and (+) α-pinene, but group 1 was less variable overall and had a lower (−):(+) α-pinene ratio than group 2. These enantiomeric phenotypes were maintained in both natural and provenance stands, were both prominent across jack pine's range, existed at the same sites and showed no correlation to any climatic variables, thereby strongly demonstrating genetic control of the trait. These α-pinene groups may be important to bark beetles, including invasive *D. ponderosae* as bark beetle pheromone production pathways that depend on α-pinene can be enantioselective (Klimetzek and Francke, 1980; Gries et al., 1990; Blomquist et al., 2010; Erbilgin et al., 2014). This may, in turn, represent a genetic basis for the suitability of individual jack pines for bark beetle colonization.

### Concluding remarks

Variations in jack pine's monoterpene composition are influenced by both genetic and environmental factors and this variability is expected to have cascading impacts on attacking herbivorous insects. While the impacts of our findings on bark beetles should be interpreted cautiously, as monoterpene composition varies between tissues of an individual tree (Latta et al., 2000; Erbilgin and Colgan, 2012) there is reason to believe that our defined jack pine chemotypes will have important implications for the biology and ecology of *D. ponderosae* in the jack pine boreal forest. The beetle may preferentially colonize α-pinene chemotype trees as α-pinene is a direct precursor of the female beetle's primary aggregation pheromone, *trans*-verbenol (Blomquist et al., 2010; Erbilgin et al., 2014). This pheromone is essential for attraction of mates and successful aggregation to overwhelm host tree defenses and beetles produce more *trans*-verbenol in host trees that have higher α-pinene concentrations leading to increased *D. ponderosae* attraction (Pitman et al., 1968; Gries et al., 1990; Pureswaran et al., 2000; Safranyik et al., 2010; Erbilgin et al., 2014). Conversely, limonene is known to be particularly toxic to *D. ponderosae* and its associated fungi (Raffa and Berryman, 1983; Clark et al., 2014) and therefore limonene chemotype trees may have detrimental effects on beetle colonization. However, because these chemotypes persist as heterogeneous stands, areas of increased susceptibility or resistance to *D. ponderosae* on a landscape scale are not expected. At this point, how jack pine's diverse phytochemical landscape will affect *D. ponderosae*'s continued eastward spread is difficult to predict, though it should be further studied, as it will likely be an important factor for the beetle's overall success in its novel host ecosystem. Additionally, as certain monoterpenes are correlated to climate variables while others are not, climate's effect on monoterpene composition could alter jack pine's susceptibility to numerous insect herbivores and pathogens, as monoterpenes affect attacking guilds in different ways (Raffa et al., 2005; Colgan and Erbilgin, 2010). This information should be considered in the context of a changing climate and large implications of the impact of climate change on plant monoterpene composition should not be overlooked.

### Conflict of interest statement

The authors declare that the research was conducted in the absence of any commercial or financial relationships that could be construed as a potential conflict of interest.
