# Supplementary material for: Variations in foliar monoterpenes across the range of jack pine reveal three widespread chemotypes: implications to host expansion of invasive mountain pine beetle
Source: Front Plant Sci. 2015 May 19;6:342. doi: 10.3389/fpls.2015.00342 (PMC4436562; doi:10.3389/fpls.2015.00342)
Supplement: Supplementary file 2 [file Table2.DOCX]

Supplementary Table 2. Pearson correlation coefficient values (r) between climatic variables and individual monoterpene concentrations in jack pine (Pinus banksiana) provenance stands. MAT=Mean Average Temperature, AWT=Average Winter Temperature, AST=Average Summer Temperature, TD=Continentality (Temperature Differance between warmest and coldest month), MAP=Mean Annual Precipitation, DD0=Degree-Days above 0°C.

|  | **Longitude** | **MAT** | **AWT** | **AST** | **TD** | **MAP** | **DD0** |
| --- | --- | --- | --- | --- | --- | --- | --- |
| **(-)-α-Pinene** | 0.319 | -0.0034 | 0.0171 | 0.00615 | -0.008 | 0.223 | -0.0109 |
| **(+)-α-Pinene** | 0.213 | -0.0217 | -0.0493 | 0.0653 | 0.078 | 0.155 | 0.0474 |
| **(-)-β-Pinene** | -0.227 | -0.204 | -0.271 | 0.0525 | 0.258 | -0.226 | 0.272 |
| **(+)-β-Pinene** | -0.23 | -0.235 | -0.288 | 0.0281 | 0.259 | -0.231 | 0.292 |
| **(-)-Limonene** | 0.152 | -0.18 | -0.214 | 0.0381 | 0.197 | 0.15 | 0.226 |
| **(+)-Limonene** | 0.121 | -0.181 | -0.237 | 0.0582 | 0.225 | 0.143 | 0.243 |
| **3 Carene** | 0.252 | -0.127 | -0.0154 | -0.3798 | -0.153 | 0.327 | 0.0535 |
| **Myrcene** | 0.349 | -0.0974 | 0.0319 | -0.2932 | -0.173 | 0.367* | -0.00545 |
| **Terpinolene** | 0.237 | 0.0924 | 0.118 | -0.0012 | -0.105 | 0.443* | -0.106 |
