# Supplementary material for: Variations in foliar monoterpenes across the range of jack pine reveal three widespread chemotypes: implications to host expansion of invasive mountain pine beetle
Source: Front Plant Sci. 2015 May 19;6:342. doi: 10.3389/fpls.2015.00342 (PMC4436562; doi:10.3389/fpls.2015.00342)
Supplement: Supplementary file 3 [file Image1.PDF]

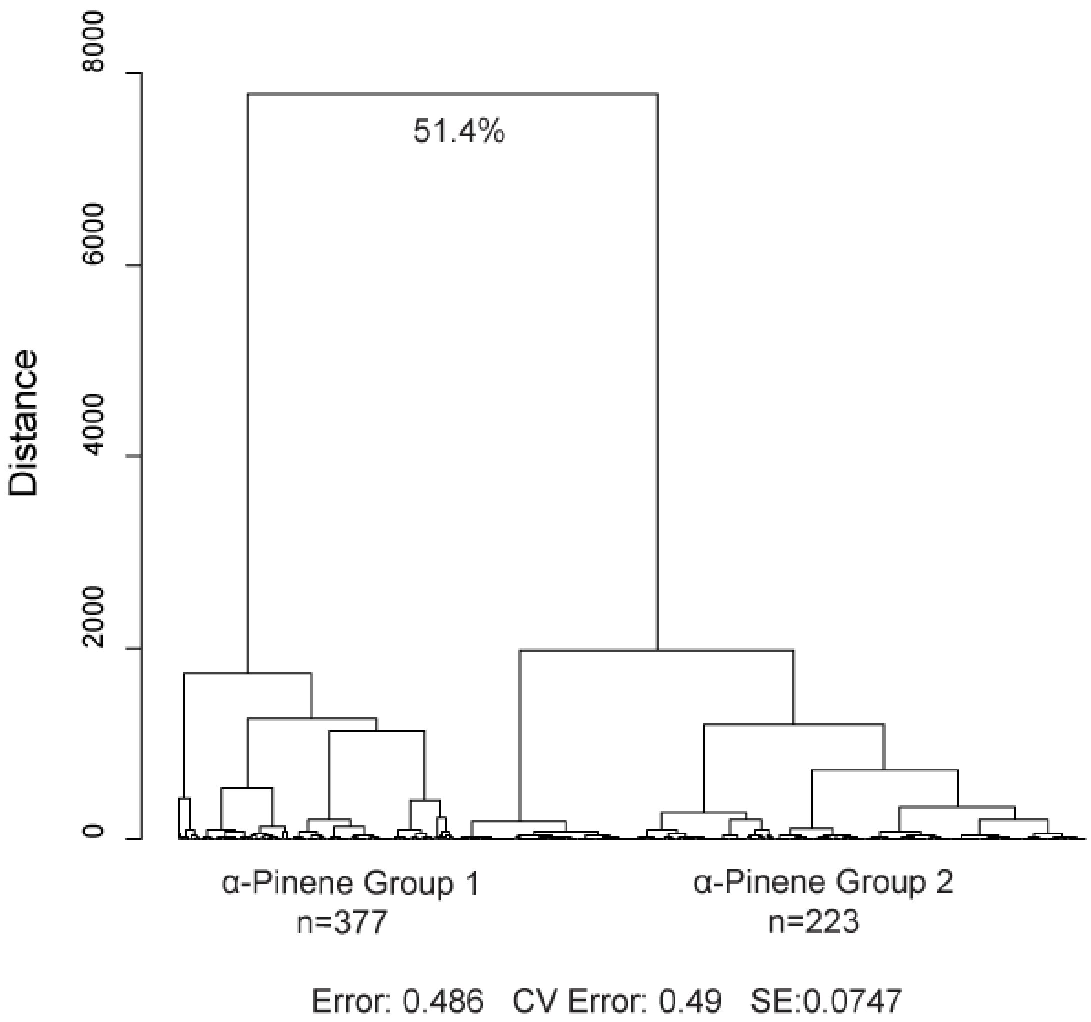

Image 1: Hierarchical cluster analyses of (-):(+) α-pinene in jack pine (*Pinus banksiana*) classifying trees into two phenotypes based on the broadest separation of enantiomeric ratios of (-):(+) α-pinene. The percent value at the first division represents the variance it explained, the error is the remaining variance not explained by the first division, CV error is the cross validated error and SE is the standard error.
